# Advances in the Exploration of Coordination Complexes of Vanadium in the Realm of Alzheimer’s Disease: A Mini Review

**DOI:** 10.3390/molecules30122547

**Published:** 2025-06-11

**Authors:** Jesús Antonio Cruz-Navarro, Luis Humberto Delgado-Rangel, Ricardo Malpica-Calderón, Arturo T. Sánchez-Mora, Hugo Ponce-Bolaños, Andrés Felipe González-Oñate, Jorge Alí-Torres, Raúl Colorado-Peralta, Daniel Canseco-Gonzalez, Viviana Reyes-Márquez, David Morales-Morales

**Affiliations:** 1 Centro de Investigación en Materiales Avanzados S.C. (CIMAV), Miguel de Cervantes No. 120, Complejo Industrial Chihuahua, Chihuahua C.P. 31136, Mexico; 2 Instituto de Química, Universidad Nacional Autónoma de México, Circuito Exterior S/N, Ciudad Universitaria, Alcaldía Coyoacán, Ciudad de México C.P. 04510, Mexico; luis.delgado@iquimica.unam.mx (L.H.D.-R.); rmalca96@comunidad.unam.mx (R.M.-C.); sanchez_atsm@hotmail.com (A.T.S.-M.); 421073867@quimica.unam.mx (H.P.-B.); 3 Departamento de Química, Universidad Nacional de Colombia-Sede Bogotá, Bogotá 111321, Colombiajialit@unal.edu.co (J.A.-T.); 4 Facultad de Ciencias Químicas, Universidad Veracruzana, Prolongación de Oriente 6, No. 1009, Col. Rafael Alvarado, Orizaba, Veracruz C.P. 94340, Mexico; racolorado@uv.mx; 5 SECIHTI-Laboratorio Nacional de Investigación y Servicio Agroalimentario y Forestal, Universidad Autónoma Chapingo Texcoco de Mora, Estado de Mexico C.P. 56230, Mexico; 6 Departamento de Ciencias Químico-Biológicas, Universidad de Sonora, Luis Encinas y Rosales S/N, Hermosillo, Sonora C.P. 83000, Mexico

**Keywords:** Alzheimer’s disease, vanadium complexes, metallodrugs, neurodegenerative disorder, oxovanadium (IV), peroxovanadium (V)

## Abstract

Alzheimer’s disease (AD) is a progressive neurodegenerative disorder characterized by cognitive decline, memory loss and limited therapeutic options. Metal-based drugs have emerged as promising alternatives in the search for effective treatments, and vanadium coordination complexes have shown significant potential due to their neuroprotective and anti-aggregant properties. This review explores the advances in the development of vanadium-based metallodrugs for AD, focusing on their ability to modulate amyloid-beta (Aβ) aggregation, oxidative stress, and neuroinflammation. Recent in vitro and in vivo studies highlight the efficacy of oxovanadium (IV) and peroxovanadium (V) complexes in inhibiting Aβ fibril formation and reducing neuronal toxicity. Additionally, the interaction of vanadium complexes with key biological targets, such as peroxisome proliferator-activated receptor gamma (PPARγ) and protein-tyrosine phosphatase 1B (PTP1B), suggests a multifaceted therapeutic approach. While these findings underscore the potential of vanadium compounds as innovative treatments for AD, further research is needed to optimize their bioavailability, selectivity, and safety for clinical applications.

## 1. Introduction

**Figure 1:**
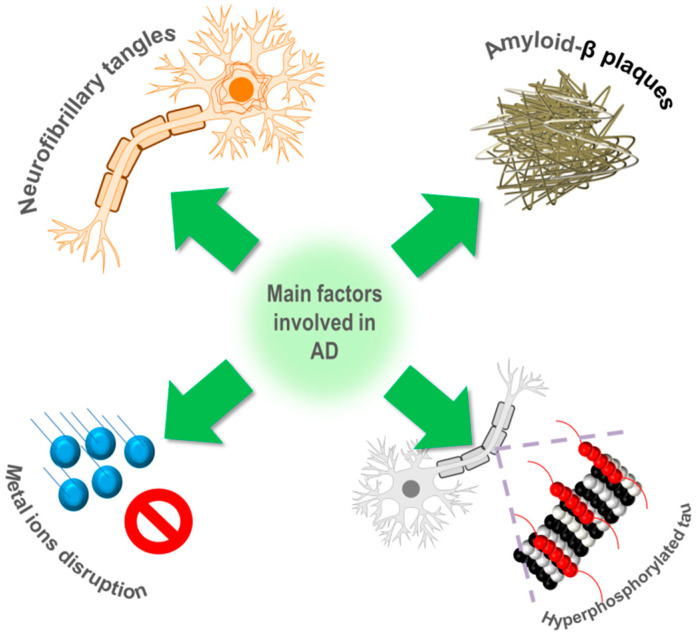
Diverse factors that cause AD.

Alzheimer’s disease (AD) represents one of the most pervasive and debilitating neurodegenerative disorders, characterized by progressive cognitive decline, memory loss, and behavioral changes [1,2,3]. With increasing prevalence due to the aging of the global population, AD poses a significant challenge to public health systems worldwide [4,5,6]. Since its first report in the early 20th century, several molecular pathways have been proposed to understand the mechanisms related to AD. In this respect, at the present time, it is known that the pathology underlying AD is multifactorial (Figure 1), involving the aggregation of amyloid-beta (Aβ) peptides [2,7], the presence of neurofibrillary tangles [7], hyperphosphorylated *tau* [8], and disruptions in metal ion homeostasis [9]. Current therapeutic strategies for AD primarily involve the use of cholinesterase inhibitors that prevent the breakdown of acetylcholine [10,11]. However, as the disease progresses, these treatments lose their effectiveness [12]. Consequently, the search for new treatment strategies, such as the development and application of metallodrugs, has gained significant attention in recent years as a promising way to combat this degenerative condition.

The field of application of metal complexes as metallodrugs has demonstrated the interesting and selective antitumor properties of several transition metal complexes such as zinc, palladium or platinum, whereas vanadium complexes have garnered significant attention for their potential as therapeutic agents in addressing several critical health challenges [13]. Research has focused mainly on their applications in various biological activities, including their anticancer, anti-inflammatory, and insulin-mimetic properties, making them promising candidates for innovative medical interventions [14].

On the other hand, oxovanadium (IV) complexes have emerged as promising metallodrugs in the search for effective treatments for AD. A growing body of evidence highlights the neuroprotective properties of these coordination complexes in both in vitro and in vivo AD models. Diverse oxovanadium (IV) complexes have been shown to inhibit Aβ aggregation and reduce oxidative damage, which are key elements in mitigating the progression of neurodegeneration. These properties have placed vanadium coordination complexes in a valuable position as attractive scaffolds for developing therapeutic agents tailored to the complex pathology of AD.

Additionally, their structural versatility enables the incorporation of ligands that enhance their stability, solubility, and selectivity, allowing for targeted delivery to the central nervous system (CNS) [13]. Innovations in ligand design, including bioactive molecules and functionalized carriers, have further broadened the therapeutic potential of vanadium complexes, offering opportunities to improve their pharmacokinetic and pharmacodynamic profiles.

This review aims to provide a comprehensive analysis of the advances in the application of oxovanadium complexes in AD research. We begin by exploring the unique chemical and biological properties of vanadium that make it particularly suitable for therapeutic applications in neurodegenerative disorders. The discussion then transitions to an overview of the main aspects related to the Aβ plaque theory and, finally, to recent breakthroughs in vanadium-based metallodrugs for the treatment of AD, highlighting their impact on enhancing the efficacy and safety of these coordination complexes. Finally, we address the challenges and limitations associated with the clinical translation of vanadium-based therapeutics, along with perspectives on future research directions in this exciting field.

## 2. Coordination Chemistry and Biological Properties of Vanadium

Vanadium is a biologically significant trace element characterized by its diverse oxidation states, ranging from +2 to +5 [15]. This element can be found in diverse cationic and anionic forms such as vanadyl cation (VO)^+2^, metavanadate (VO_3_)^−^ and orthovanadate (VO_4_)^−3^. In the intracellular medium, vanadium exists primarily in oxidation states such as +3, +4, and +5. This redox flexibility is key to its interaction with biomolecules, enabling it to influence enzymatic activity, regulate metabolic pathways, and facilitate electron transfer reactions [12,16].

**Figure 2:**
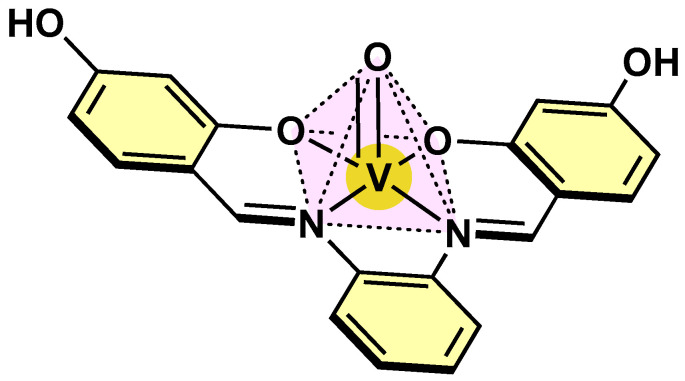
Square pyramidal geometry of an oxovanadium (IV) complex derived from Schiff bases.

Vanadium coordination chemistry and its related geometry depend on its oxidation number. For oxovanadium (IV) moieties, a coordination number of five is typically observed in most of its complexes. In this respect, the pentacoordinated complexes present a square pyramidal geometry, with the oxygen atom in the apical position of the oxovanadium (IV) unit (Figure 2). In this arrangement, the vanadium atom is positioned above the equatorial plane, which is formed by the four donor atoms of the equatorial ligands [17]. In the case of hexacoordinated structures, the geometry adopted is typically a distorted octahedron. For complexes having a vanadium (V) center, they possess a variety of geometries ranging from tetrahedral, pentagonal-bipyramidal and octahedral [18].

In biological systems, the structural similarities of the coordination environment of vanadium (IV) with phosphate ion (PO_4_)^3−^ comprise the key factor interfering in the ATP phosphorylation/dephosphorylation route in glucose or lipid metabolism. This property allows vanadium complexes to influence signalling pathways and enzymatic reactions. Therefore, in recent years, vanadium complexes have been studied for their potential application in treating different diseases such as cancer, diabetes and AD. The selection of the ligand for vanadium complexes further improves their medicinal effects and their selectivity towards the desired target. In this regard, vanadium complexes are easily absorbed in the oxidation states described above, and when they reach the bloodstream, they are transported mainly by serum proteins [19]. Histidine (His) and cysteine (Cys) residues are the main coordination sites for vanadium in several proteins, which gives them the ability to mimic and substitute phosphate groups [20]. Unlike vanadyl compounds, phosphates cannot have a stable penta- or hexacoordinated form with proteins.

This coordination in phosphate derivatives is only a transition state; in contrast, oxovanadium compounds promote a stable coordination, which has sparked significant interest in their potential applications for the management of metabolic disorders such as diabetes, where the strong coordination with Cys residues in tyrosine phosphatase avoids dephosphorylation of the insulin receptor [21,22,23]. A phosphorylated insulin receptor promotes glucose uptake into the cell; consequently, serum glucose levels decrease. Many examples of oxovanadium complexes have been used and tested in humans and rats; most of them are derived from vanadyl with natural products [19,21,22,23,24].

In addition to mimicking phosphate in biological processes, vanadium (V) complexes can interact with proteins and other cell structures. The potential therapeutic application of peroxovanadium (V) complexes in AD is due to their ability to modulate Aβ aggregation by selectively oxidizing methionine-35, thereby preventing the formation of toxic Aβ plaques. These plaques play a critical role in the pathogenesis of AD, as their accumulation leads to neuronal dysfunction and cognitive decline.

## 3. Amyloid-Beta Plaques in Alzheimer’s Disease

Aβ is a peptide fragment derived from the enzymatic cleavage of amyloid precursor protein (APP), a transmembrane glycoprotein abundantly expressed in the brain [24,25,26]. It is possible to find peptide fragments of Aβ made up of 38 and up to 43 amino acid units, the most common being Aβ-40, which does not represent a direct health risk [27,28,29,30,31,32,33,34,35,36,37,38,39,40,41]. Even under physiological conditions, Aβ plays a role in several cellular processes, including signal transduction, synaptic plasticity, and neuronal repair [36,40]. In this context, the Aβ-42 peptide is recognized as the main factor responsible for the formation of so-called senile plaques, and the ratio between Aβ-40 and Aβ-42 is often monitored, with an increase in Aβ-42 being considered risky [27,28,29,30,31,32,33,34,35,36,37,38,39,40,41]. These plaques are primarily formed by Aβ peptides, which are commonly 40 to 42 amino acids long and typically contain a hydrophobic segment that decreases their solubility, promoting aggregation, along with a region (D1-K16) that exhibits a strong affinity for transition-metal cations [34,35,36,37,38,39,40,41].

**Figure 3:**
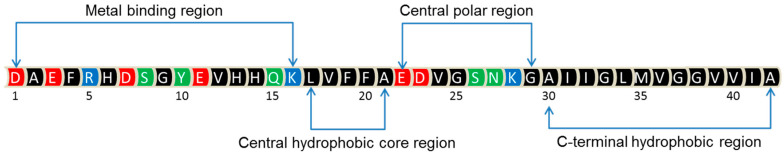
The primary structure of Aβ-42. The polar residues are highlighted in green, the acidid residues in red, the basic residues in blue, and the hydrophobic residues in black. Redapted from reference [35] with permission of Elsevier.

As described by Li et al. the typical structural composition of Aβ-42 consists of four parts: a metal-binding region, a central hydrophobic core (CHC), a central hydrophilic region, and a C-terminal hydrophobic region [27,35] (Figure 3). On the one hand, as described by Owen et al., the metal-binding region is chemically uninteresting, as it turns out to have a high affinity for divalent cations such as zinc(II) and copper(II), as well as the C-terminal hydrophobic region, which compared to Aβ-40 only has two extra amino acids (isoleucine, Ile, I, and alanine, Ala, A), which are enough to increase its hydrophobicity, causing aggregates of this fragment to form, thus increasing its toxicity [35].

The study of Aβ is of clinical importance since, as the “amyloid cascade hypothesis” explains, the accumulation of Aβ leads to the formation of senile plaques, which are Aβ aggregates, into higher-order oligomers and fibrils, which can cause effects on cellular function, including alteration of synaptic activity and synapse loss, alteration of cerebral capillary blood flow, and direct promotion of *tau* pathology by stimulating *tau* hyper-phosphorylation [34]. The “amyloid cascade hypothesis”, along with *tau* hyperphosphorylation, are considered the main causes of AD [42].

**Figure 4:**
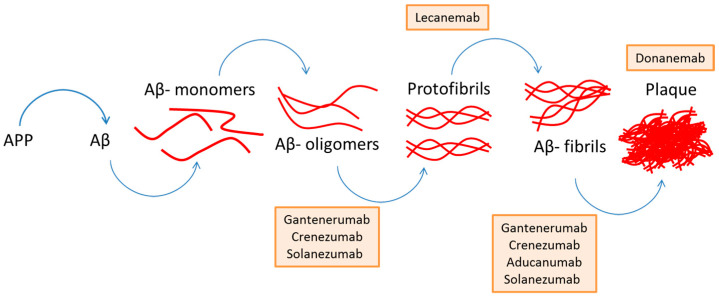
General diagram of the amyloid cascade, from APP to plaque formation, indicating some of the drugs used for AD and the part of the cascade where they intervene. Adapted from reference [46].

Aβ aggregation is thought to be a response to other underlying pathological mechanisms, such as oxidative stress, neuroinflammation, diabetes, alterations in cholesterol homeostasis, dysfunction in mitochondrial activity, metal ion regulation, etc. [43,44]. Nonetheless, Aβ aggregation is one of the leading causes of AD, so many treatments target this peptide [43,44,45]. Several of the treatments target the hydrophobic regions of Aβ, intervening in the formation of Aβ aggregates such as Aβ-oligomers, protofibrils, Aβ fibrils or in the formation of plaques [46], as described in Figure 4. This is where some of the drugs currently used are indicated, as well as the part of the amyloid cascade in which they intervene. Additionally, recent failures of Aβ-targeting therapies, including monoclonal antibodies such as solanezumab and aducanumab, have raised questions about the effectiveness of therapies targeting Aβ alone in AD treatment.

Given the complexity of AD, researchers have explored alternative approaches to inhibit Aβ aggregation. Among them, vanadium-based compounds have emerged as promising candidates due to their ability to selectively oxidize the methionine-35 residue of Aβ, preventing its aggregation into neurotoxic plaques.

Recent studies have demonstrated that peroxovanadium (V) complexes can modulate Aβ aggregation through targeted oxidation, effectively reducing plaque formation and its associated neurotoxicity. Although these advances have been explored primarily in vitro and cell cultures, they open new avenues for the development of metal-based therapeutic strategies aimed at mitigating Aβ-induced neurodegeneration.

## 4. Recent Advances

### In Vitro and In Vivo Evaluation of Vanadium Complexes

At present, several vanadium complexes based on important ligands have been successfully evaluated in vitro and in vivo as Aβ inhibitors. In this regard, He et al. reported two peroxovanadium (V) complexes, **1** and **2**, which were tested against the formation of amyloid-β (Aβ) and prion-protein (PrP) deposits [46]. In an in vitro study, **1** and **2** were found to inhibit the formation of large aggregates of these proteins in a dose-dependent manner. These complexes exerted their inhibitory properties by oxidizing His111 and Met109/112 in PrP and Met35 in Aβ, which are crucial for forming large aggregates. A detailed proposed mechanism of action for peroxovanadium complexes involves the oxidation of key residues in β-amyloid plaques, particularly methionine. These vanadium complexes appear to interact directly with amyloid peptides through coordination with histidine and methionine residues, subsequently oxidizing methionine to methionine sulfoxide. This oxidation disrupts the secondary structure of the peptides and delays their aggregation.

This mechanism [47] has been supported by data from NMR, circular dichroism (CD) spectroscopy, mass spectrometry, dynamic light scattering (DLS), atomic force microscopy (AFM), and fluorescence spectroscopy. Additionally, cell viability assays in human neuroblastoma cells indicate increased viability upon treatment with vanadium complexes, suggesting a protective effect.

These kinds of complexes have been also tested in a cell proliferation test in the presence of Aβ and PrP aggregates, where they augmented the percentage of neuroblastoma cell proliferation. Although a therapeutic effect was observed, the possibility of cytotoxicity presented by **1** and **2** was not ruled out.

Because Aβ plaque formation is one of the main pathologies leading to AD, Tan et al. tested the ability of vanadyl (IV) acetylacetonate (VAC) (**3**) to inhibit Aβ formation in vivo. The test was made in two administration groups: at three months and at five months of age. The objective of this age difference was to observe the preventive potential of **3** on neuronal damage induced by Aβ deposition. Even though a positive effect was observed, administration at three months of age was not superior compared to administration at five months. The therapeutic effect was attributed to the fact that **3** was able to activate the proliferator-activated receptor γ (PPARγ) and AMP-activated protein kinase α (AMPKα), two proteins related to glucose and energy metabolism. It was also observed that the improvement in glucose metabolism induced neuroprotection by activating glucose-regulated protein 75 (Grp75). As *tau* phosphorylation is part of AD pathology, the expression of c-Junk amino-terminal kinase (JNK) was also studied. It was found that **3** inhibited JNK phosphorylation, consequently diminishing *tau* phosphorylation [48].

Different research groups have also considered the evaluation of vanadium(IV) complexes with Schiff bases. For example, complexes **4**, **5**, **6** and **7** were reported by Xu et al. [49]. These compounds were tested against the aggregation of human islet amyloid polypeptide (hIAPP) in vitro and in vivo. hIAPP is a peptide produced by the pancreas in insulin secretion, which can misfold and contribute to AD pathology. Administration of **4**–**7** into cell cultures with pre- and post-aggregation of hIAPP showed that all four compounds could shorten the size of hIAPP aggregates but could not prevent aggregate formation. In vivo studies confirmed the aggregation inhibition behavior presented in vitro, with **4** being the most effective. Free ligands were also tested and found to have inhibitory properties but to a lesser extent than the complexes.

These findings remark on the importance of the vanadium metal center for this type of compound to exert its therapeutic effects. Xu et al. also reported the ability of **1** and two other vanadium complexes, BEOV (**8**) and the binuclear vanadium (V) complex (**9**), to inhibit hIAPP aggregation [50]. The inhibitory effects were tested against the full-length hIAPP protein and its fragments, hIAPP_20–29_ and hIAPP_19–37_, which are involved in insulin binding and amyloid deposit formation, respectively. It was observed that **1**, **8** and **9** have inhibitory activity on aggregate formation in a dose-dependent manner, with **3** being the most effective.

A delay in hIAPP aggregation was also observed, implying that all three complexes not only diminished the size of the aggregates but also augmented the time taken for them to form. By measuring thermodynamic values, it was found that these complexes interacted mainly with hIAPP and its fragments through hydrophobic interactions. Based on these measurements, it was implied that the superior activity of **9** was due to more aromatic moieties, followed by **8** and **1** in that order. To conclude this study, an in vitro cell proliferation assay was performed using rat insulinoma cells (INS-1) in the presence of hIAPP aggregates. The aggregates induce significant cytotoxicity, with hIAPP being the most toxic. The complexes showed a beneficial effect by increasing the proliferation of INS-1 cells. However, the cytotoxicity of the vanadium complexes incubated without hIAPP was appreciable. Nevertheless, when applied to cell culture with hIAPP, it was found that **1**, **8**, and **9** were able to alleviate the toxicity of the aggregates by decreasing their number and size.

In a series of three papers [51,52,53], He et al. explored the interaction of complex **8** (also known as BEOV) and PPARγ and its therapeutic implications in AD in vitro and in vivo. First, the therapeutic effect of BEOV against endoplasmic reticulum (ER) stress was studied in vitro. For this purpose, hippocampal and neuroblastoma cells were cultured in the presence of Aβ aggregates, and the expression of ER stress proteins of the glucose-regulated protein 78 (Grp78) pathways was measured. Furthermore, pro-apoptotic proteins of the Grp78 pathway were expressed in cell culture with Aβ deposits. When complex **8** was cultured together with Aβ, it was found to exert a dose-dependent therapeutic effect, which decreased the expression levels of Grp78 pro-apoptotic proteins and increased the expression of PPARγ, thereby increasing cell viability. PPARγ is considered to be an ER stress regulator. Hence, the same principle was tested in vivo in APP/PS1 transgenic mice. BEOV prevented apoptotic cell death, which was confirmed by measuring the expression levels of pro-apoptotic proteins. To better correlate the therapeutic effect of BEOV with PPARγ interaction, a PPARγ inhibitor was used in conjunction with a BEOV dose. Inhibition nullified the positive impact on cell viability, confirming the agonistic properties of BEOV. Later, employing a triple transgenic mice model (3xTg-AD), the effect of the interaction of BEOV with PPARγ preventing neuronal damage caused by Aβ deposits was studied in vivo. β-secretase (BACE1) is the primary Aβ protein precursor in AD pathology and is regulated by PPARγ.

**Figure 5:**
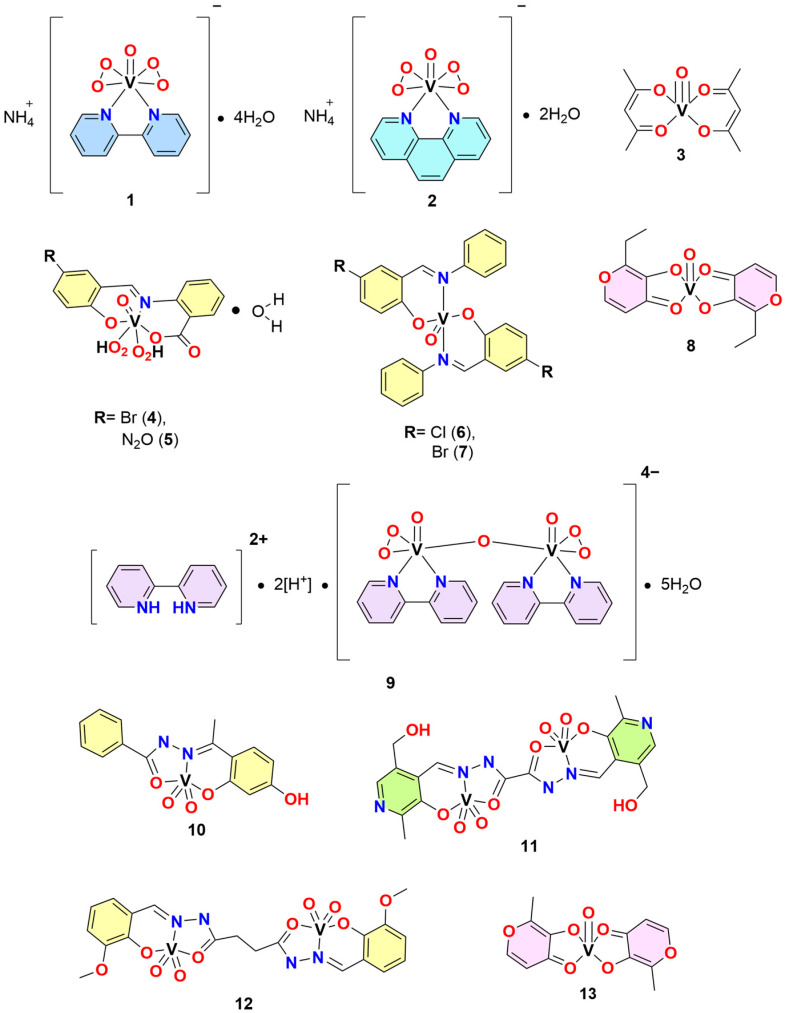
Molecular structure of diverse vanadium complexes.

BEOV administration showed a decrease in Aβ deposits, a decrease in BACE1 activity, and an increase in PPARγ activity. Employing a cognitive function test, BEOV was found to prevent cognitive function loss in a dose-dependent manner. It also increased neuronal density in the brains of mice. Due to the relationship between diabetes and AD, glucose uptake was also measured through radioactive emission. BEOV was able to normalize glucose intake in the brains of AD-affected mice and regulate insulin receptor (InsR) activity, a vital intermediary in Aβ-induced insulin resistance. Together with the inhibition of BACE1 activity, BEOV prevented *tau* phosphorylation by targeting two of the main pathologies of AD [52] (Figure 5).

On the other hand, the regulation of the inflammatory response of microglia triggered by Aβ was studied in vitro and in vivo. Microglia are a type of immune system cell that surrounds Aβ plaques when activated. After activation, they release inflammatory-inducing proteins from the nuclear factor-kappa B (NF-κB) pathway, resulting in an inflammatory response leading to neuronal death. PPARγ is a regulator protein of the inflammatory response, which makes it a good target to ameliorate the damage caused by inflammatory cell death. When microglia cells were cultured in the presence of Aβ, and the brains of APPswe/PS1E9 mice were studied, a pro-inflammatory response was observed. The expression of NF-κB pathway proteins was increased. After BEOV administration, the expression of NF-κB and its pro-inflammatory proteins decreased to normal levels, while the expression of PPARγ was increased. To confirm this effect, a PPARγ inhibitory test was performed in the same manner mentioned previously [53]. Inhibition of PPARγ eliminated the therapeutic impact of BEOV and increased the expression of NF-κB and its pro-inflammatory proteins. This series of studies highlights the relevance of the interaction of vanadium complexes, specifically complex 8 with PPARγ, in developing new metallodrugs against AD. As part of this section, the biological activity of complexes 1–9 is summarized in Table 1.

Conjointly with the experimental studies, computational investigations contribute to the understanding of the molecular interaction between vanadium complexes and biomolecules of relevance in AD. Chaves et al. reported on the molecular docking of **10**, **11** and **12**, using the GOLD5.5 software, with the acetylcholinesterase enzyme (AChE). AChE is an enzyme that activates neuronal communication through acetylcholine [54]. Mild inhibition or activation of AChE has been shown to have therapeutic effects against AD. Experimentally, **10** activated AChE by 47.0%, while **11** and **12** inhibited it by 20.0% and 21.9%, respectively. In molecular docking, all three complexes were found to interact with AChE through hydrogen bonds, Van der Waals interactions, and hydrophobic interactions.

**Figure 6:**
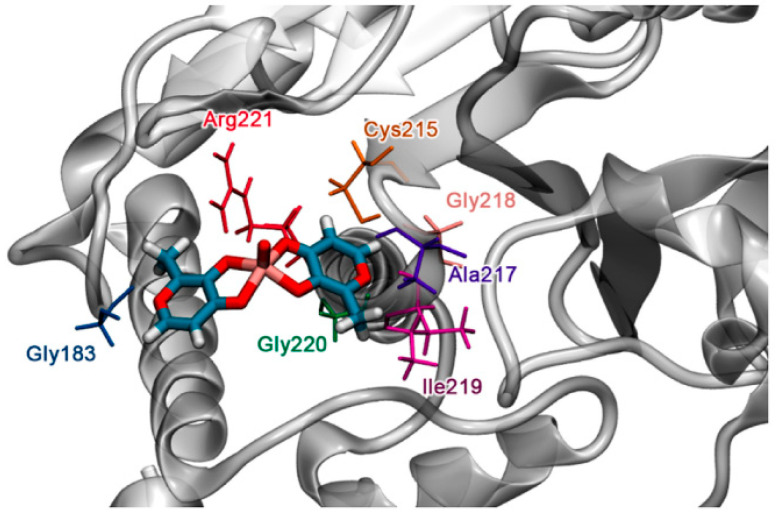
Molecular docking of the BMOV-PTP1B system. Reproduced from reference [55] with permission of Elsevier.

On the other hand, Tavares et al. developed a new force field in the Assisted Model Building with Energy Refinement (AMBER) software to study the interaction between the vanadium (IV) complex (**13**) and protein-tyrosine phosphatase 1B (PTP1B) [55]. To assess the accuracy of AMBER modelling, experimental structural data of **13** (bond angles and bond distances) were compared with data obtained with the new force field. Once the basis set was optimized, docking experiments were performed to obtain the lowest energy interaction between **13** and PTP1B. Using as an anchor the interactions already reported experimentally between **13** and Gly183 and Arg221, it was found that the complex interacted with Gly183, Cys215, Ala217, Gly218, Ile219, Gly220, and Arg221 with a minimum energy of −104.811 kcal mol^−1^ (Figure 6). These findings highlight the importance of the computational development of methodologies capable of generating prediction and acquisition of appropriate data to better understand the interaction and therapeutic effects of vanadium compounds against biomolecules of relevance in AD.

## 5. Outlook and Perspectives

Research on vanadium-based complexes as potential therapeutic agents for AD has demonstrated promising outcomes in both in vitro and in vivo studies. The ability of these complexes to inhibit Aβ and PrP aggregates, as well as their role in modulating key biochemical pathways involved in neurodegeneration, underscores their potential as metallodrugs. Notably, oxidation of critical residues in PrP and Aβ, activation of PPARγ, and modulation of AMPKα contribute to their therapeutic effects. Additionally, Schiff base-derived vanadium (IV) complexes have shown significant inhibitory activity against hIAPP aggregation, which links metabolic disorders to neurodegeneration.

A crucial finding from these studies is the importance of the vanadium metal center in mediating therapeutic activity, reinforcing the need for further structural optimization of these compounds. The results of BEOV highlight its role in preventing neuronal damage through its interaction with PPARγ, a key regulator of metabolic and inflammatory pathways. In vivo experiments using APP/PS1 and 3xTg-AD transgenic mouse models revealed that BEOV administration significantly reduced Aβ deposits, improved glucose metabolism, and prevented *tau* phosphorylation—key hallmarks of AD pathology. These findings establish a strong rationale for advancing BEOV and other similar vanadium complexes into preclinical and clinical trials.

Despite the encouraging results, some challenges remain to be resolved. One of the primary concerns is the potential cytotoxicity of these vanadium complexes when administered without the presence of amyloid aggregates. Future research should focus on refining the structural features of these compounds to enhance their selectivity and reduce unwanted toxicity. Additionally, further exploration of the bioavailability and pharmacokinetics of vanadium compounds is needed to determine their long-term safety and efficacy in human models.

Computational studies have also played a pivotal role in understanding the molecular interactions of vanadium complexes with key biomolecules implicated in AD. Molecular docking and force field optimizations have provided valuable insights into how these complexes interact with AChE and PTP1B. These computational approaches offer a powerful tool for predicting and designing new metallodrugs with improved therapeutic profiles.

Looking ahead, the integration of experimental and computational research will be essential for the rational design of next-generation vanadium-based therapeutics. Further investigations into their mechanisms of action, long-term stability, and interactions with biological systems will be crucial for their clinical translation. The ongoing exploration of vanadium complexes as treatments for AD represents an exciting frontier in metallodrug research, with the potential to address the pressing need for effective disease-modifying therapies.

## 6. Conclusions

Vanadium complexes represent an emerging class of potential metallodrugs for neurodegenerative diseases, particularly AD. Their diverse mechanisms of action—including inhibition of protein aggregation, modulation of metabolic pathways, and anti-inflammatory effects—underscore their therapeutic relevance. Notably, peroxovanadium (V) complexes, Schiff base-derived vanadium (IV) compounds, and BEOV have demonstrated promising neuroprotective properties in both in vitro and in vivo models.

However, despite these promising findings, significant hurdles remain, including cytotoxicity concerns, bioavailability, and long-term safety. The integration of computational studies has provided critical insights into molecular interactions, paving the way for rational drug design and optimization. Further interdisciplinary research is needed to refine these complexes, enhance their selectivity, and ensure their safe application in clinical settings.

With continued advances in metallodrug development, vanadium-based compounds have the potential to contribute significantly to the ongoing search for effective treatments against AD and related neurodegenerative disorders.

## Figures and Tables

**Table 1:** Summary of biological activity of diverse vanadium complexes evaluated as AD metallodrugs.

Complex	Oxidation Statate	Activity Model	Biological Activity Summary	Reference
**1**	+5	In vitro	Inhibits Aβ and PrP aggregation via oxidation of His111/Met residues; increases neuroblastoma cell viability	[47]
**2**	+5	In vitro	Similar to 1; methionine oxidation delays aggregation	[47]
**3**	+4	In vivo	Activates PPARγ and AMPKα; reduces Aβ deposition and *tau* phosphorylation; improves glucose metabolism	[48]
**4**–**7**	+4	In vitro/in vivo	Inhibit hIAPP aggregation; 4 most effective; ligands less active alone	[49]
**8**	+4	In vitro/in vivo	Interacts with PPARγ; reduces ER stress, Aβ and *tau* pathology, and neuroinflammation; improves cognition	[50,51,52,53]
**9**	+5	In vitro	Effective inhibitor of hIAPP aggregation; strongest hydrophobic interactions among tested compounds	[50]
**10**–**12**	+5	In sillico	Modulate AChE activity (activation by 10; inhibition by 11 and 12); interact via H-bonding and hydrophobic forces	[54]
**13**	+4	In sillico	Interacts with PTP1B enzyme (via Gly183, Cys215, Arg221); strong binding energy (−104.8 kcal/mol)	[55]

## Data Availability

Additional data to those shown in this review can be obtained from the articles cited in the reference list.

## Abbreviations

The following abbreviations are used in this manuscript:

Aβ	Amyloid-β
AChE	Acetylcholinesterase enzyme
AD	Alzheimer’s disease
Ala	Alanine
AMBER	Assisted Model Building with Energy Refinement
AMPKα	AMP-activated protein kinase α
Arg	Arginine
BACE1	β-secretase
BEOV	bis(ethyl-maltolato)oxido-vanadium(IV)
Cys	Cysteine
ER	Endoplasmic reticulum
Gly	Glycine
Grp75	Glucose-regulated protein 75
Grp78	Glucose regulated protein 78
Hiapp	Human islet amyloid polypeptide
His	Histidine
InsR	Insulin receptor
INS-1	Rat insulinoma
Ile	Isoleucine
JNK	c-Junk amino-terminal kinase
Met	Methionine
NF-κB	Nuclear factor-kappa B
PPARγ	Proliferator-activated receptor γ
PrP	Prion-protein
PTP1B	Protein-tyrosine phosphatase 1B
VAC	Vanadyl acetylacetonate
